# Multiplexed imaging and automated signal quantification in formalin-fixed paraffin-embedded tissues by ChipCytometry

**DOI:** 10.1016/j.crmeth.2021.100104

**Published:** 2021-10-27

**Authors:** Sebastian Jarosch, Jan Köhlen, Rim S.J. Sarker, Katja Steiger, Klaus-Peter Janssen, Arne Christians, Christian Hennig, Ernst Holler, Elvira D'Ippolito, Dirk H. Busch

**Affiliations:** 1 Institute for Medical Microbiology, Immunology and Hygiene, Technical University of Munich (TUM), 81675 Munich, Germany; 2 Comparative Experimental Pathology, Institute for Pathology, Technical University of Munich, 81675 Munich, Germany; 3 Department of Surgery, Klinikum rechts der Isar, Technical University of Munich, 81675 Munich, Germany; 4 Zellkraftwerk GmbH, 04103 Leipzig, Germany; 5 Department of Hematology/Oncology, University Medical Center, 93053 Regensburg, Germany; 6 German Center for Infection Research (DZIF), partner site Munich, 81675 Munich, Germany

**Keywords:** highly multiplexed tissue imaging, formalin-fixed paraffin-embedded samples, automated quantification of signal intensities, spatial spillover correction, cell-type segmentation

## Abstract

Deciphering the spatial composition of cells in tissues is essential for detailed understanding of biological processes in health and disease. Recent technological advances enabled the assessment of the enormous complexity of tissue-derived parameters by highly multiplexed tissue imaging (HMTI), but elaborate machinery and data analyses are required. This severely limits broad applicability of HMTI. Here we demonstrate for the first time the application of ChipCytometry technology, which has unique features for widespread use, on formalin-fixed paraffin-embedded samples, the most commonly used storage technique of clinically relevant patient specimens worldwide. The excellent staining quality permits workflows for automated quantification of signal intensities, which we further optimized to compensate signal spillover from neighboring cells. In combination with the high number of validated markers, the reported platform can be used from unbiased analyses of tissue composition to detection of phenotypically complex rare cells, and can be easily implemented in both routine research and clinical pathology.

## Highlights

• ChipCytometry is an optical imaging method based on staining/bleaching cycles
• ChipCytometry allows multiplexed imaging and is now applicable to FFPE sections
• A fine-tuned analysis pipeline leads to accurate automatic signal quantifications
• High data quality enables deep investigation of spatial and phenotypic parameters

## Motivation

Simultaneous *in situ* analyses of a multitude of cells and markers is necessary for gaining insights into tissue biology and pathology. Here we describe the application of ChipCytometry, a user-friendly optical imaging-based technology for multiplexed staining, to widespread used formalin-fixed paraffin-embedded tissues. The multiplexing of up to 30 markers in combination with a developed open-source workflow for signal quantification facilitated high-dimensional tissue analyses.

## Graphical abstract

**Figure:**
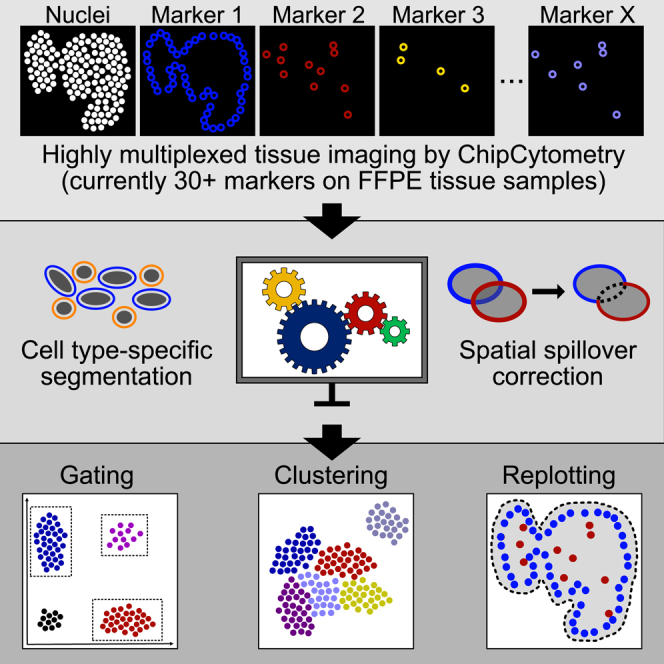

## Teaser

Understanding of tissue biology requires *in situ* analyses of cell phenotypes. Jarosch et al. describe the application of the multiplexed imaging method ChipCytometry to formalin-fixed and paraffin-embedded tissue sections. The high staining quality allowed them to develop a pipeline for automated signal quantifications facilitating reliable and reproducible analyses of high-parameter imaging.

## Introduction

Organ and tissue functions are maintained by different cell types that interact via complex networks of cell-to-cell communications. As these communications drive most physiological cellular mechanisms (Brazil et al., 2019; Fortini, 2009; Mattes and Scholpp, 2018; Rieckmann et al., 2017), imbalances in composition and/or activity of cell subtypes can disrupt the organ equilibrium and thus lead to pathological conditions. Highly multiplexed tissue imaging (HMTI) today allows the simultaneous analysis of thousands of cells with dozens of markers (Parra et al., 2019; Tan et al., 2020), thus representing a suitable method to gain deeper insights into the enormous complexity of organs and to better understand diseases (Chevrier et al., 2017; Wagner et al., 2019). Unraveling changes in cellular phenotype, tissue location, and interaction partners can ultimately translate into more tailored therapeutic interventions.

However, most of the HMTI technologies developed so far have limitations with regard to their accessibility and implementation in routine research and diagnostics. This is of utmost importance in diagnostics, where single-parameter immunohistochemistry (IHC) is still the gold-standard method. HMTI approaches (>20 markers) can be broadly classified into three categories, namely mass spectroscopy, sequencing of DNA-tagged antibodies, and optical microscopy. Imaging mass cytometry is the most used and powerful mass spectroscopy-based technique, in which tissue sections are stained with isotope-labeled antibodies and microdissected using laser ablation; microparticles are then conveyed into a mass spectrometer to finally use their time of flight to reconstruct tissue staining (Angelo et al., 2014; Giesen et al., 2014). Despite its high multiplexing, this method still requires sophisticated and expensive instruments and skills. Additionally, the tissue is destroyed after image acquisition and, depending on the resolution of laser ablation, spatial resolution might be inferior to fluorescent imaging. With the Nanostring digital spatial profiling, proteins and mRNAs can be simultaneously detected by DNA-tagged antibodies and RNA probes, respectively (Merritt et al., 2020). However, analysis of sequencing data is elaborate and single-cell resolution is lost, as DNA barcodes cannot be assigned to a specific cell but rather to a region of interest that comprises multiple cells.

Fluorescence-based methods therefore remain the best alternative for routine research and diagnostics; in particular, tissue-based cyclic immunofluorescence (t-CyCIF) and co-detection by indexing (CODEX) are gaining increasing attention as they could solve most of the above-mentioned limitations. In t-CyCIF, multiplexing is achieved by iterative cycles of staining with fluorophore-conjugated primary or secondary antibodies, image acquisition with common immunofluorescence (IF)-based microscopes, and subsequent chemical bleaching of the fluorescent dyes (Caserta et al., 2010; Gerdes et al., 2013; Lin et al., 2015, 2016, 2018). The CODEX technology is based on differently barcoded antibodies, which are detected via fluorescently labeled complementary oligonucleotides in a cyclic staining approach (Goltsev et al., 2018) (Kennedy-Darling et al., 2021). The concept of DNA-barcoded antibodies has been further elaborated by the use DNAses for specific de-staining (SeqStain) (Rajagopalan et al., 2021), and by primer exchange reactions for signal amplification (ImmunoSABER) (Saka et al., 2019).

Besides these technologies, ChipCytometry has emerged as a particularly advanced optical imaging-based platform for HMTI, expanding on the basic principles of t-CyCIF (Hennig et al., 2009), with three principal advantages: first, that images are acquired as stacks of images with different brightness (high dynamic range [HDR] imaging), which allows better resolution between low and high signal intensities; second, that HDR background is recorded before each staining and appropriately subtracted to remove autofluorescence; and third, that, remarkably, continuous operation could be ensured by fully automated instruments (Cytobot) (Zellkraftwerk). Furthermore, tissue sections might be stored for later staining, thus potentially enabling the establishment of true tissue biobanking. Overall, these features make ChipCytometry a platform highly suitable for future broad applications in clinical histopathology as well as basic research. Currently, this technology is well established for the analysis of single cells from suspensions (Teo et al., 2017) and only recently has been transferred to fresh-frozen preserved tissues (Leng et al., 2019; Mulazzani et al., 2019). However, software solutions for automated quantification of ChipCytometry tissue imaging data are not available yet. In addition, no applications have been reported so far with formalin-fixed paraffin-embedded (FFPE) tissues, although huge clinical repositories of FFPE samples exist and FFPE is still the preferred method for long-term storage in the clinic (Gaffney et al., 2018).

In this study, we demonstrate for the first time successful HMTI on clinical FFPE specimens using ChipCytometry, with a specific focus on the analysis of immune cell infiltrates in non-diseased tissues and different types of cancer sections. The high staining quality allowed the application of software tools for cell segmentation and automated signal intensities, which we further optimized for more accurate quantifications by introducing a cell-type-specific cell segmentation and a spatial spillover correction prior to signal value calculation. The quantified data could be used for both characterization of tissue composition and identification of rare but biological meaningful populations.

## Results

### Establishment of ChipCytometry for FFPE tissues

**Figure 1:**
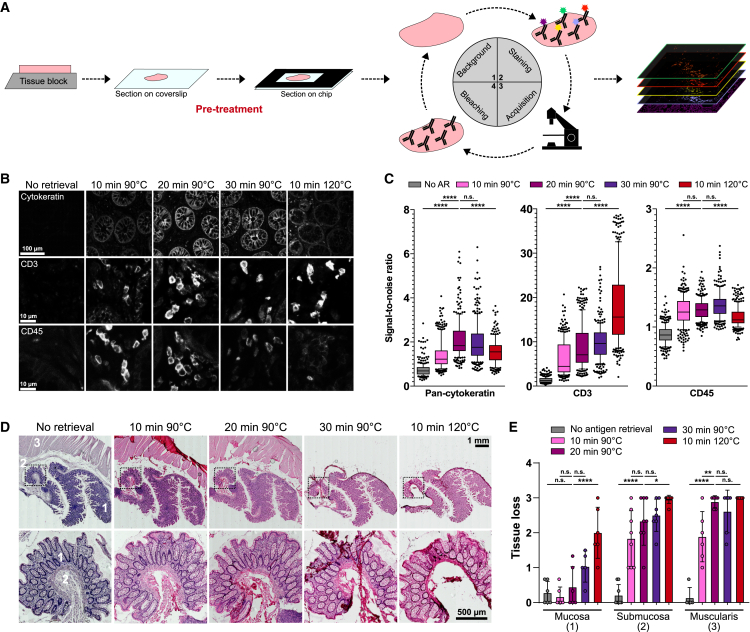
Establishment of protocols for staining of FFPE tissue sections by ChipCytometry in healthy human colon tissues (A) Schematic representation of tissue preparation and iterative staining-acquisition-bleaching cycles for highly multiplexed ChipCytometry. For FFPE tissue section, a pre-treatment for antigen retrieval is necessary before loading into the microfluidic chip. (B) Representative staining of CD3, CD45, and pan-cytokeratin according to different antigen retrieval conditions. (C) Quantification of signal-to-noise ratio for the markers shown in (B). Data are depicted as interquartile ranges, with whiskers extending to 10% and 90% and outliers plotted as dots. Individual crypts (pan-cytokeratin staining) or cells (CD3/CD45 staining) were quantified for eight positions per section. (D) Hematoxylin-eosin staining of consecutive tissue sections after different antigen retrieval treatments. Zoom in (marked area in the upper row) is shown at higher magnification in the lower panel. Numbers indicate regions of mucosa (1), submucosa (2), and muscularis (3). (E) Scoring of tissue loss (0 = no tissue integrity loss, 3 = complete loss of the tissue). Data are shown as mean ± standard deviation. In (C) and (E), significances are calculated using Tukey's test followed by Dunn's multiple comparisons test (∗p < 0.05; ∗∗p < 0.01; ∗∗∗∗p < 0.0001). A minimum of three donors in two or three independent experiments were used. See also Figures S1–S3.

For cryopreserved tissue staining, sections are mounted on glass coverslips and then loaded into a microfluidic chip. After recording of background autofluorescence, tissue sections are stained with up to five fluorophore-conjugated primary antibodies, images are acquired, and fluorophores are finally photobleached to make the tissue available for the next staining cycle (Figure 1A). The approach favors overnight antibody incubation for practical reasons, which did not show counterproductive but rather beneficial effects on staining intensities compared with shorter incubation times (Figure S1A). Iterative staining-imaging-bleaching cycles allow the detection of a theoretically unlimited number of markers at single-cell level, accomplished by a precise automatic tissue repositioning after each cycle. Alternative methods for fluorophore bleaching, such as chemical bleaching, were also compatible with the ChipCytometry platform (Figures S1B–S1D), although extensive studies on epitope preservation are required for these harsher bleaching conditions.

Unlike cryopreserved tissues, staining on FFPE sections requires an additional step of pre-treatment (i.e., antigen retrieval before incubation with primary antibodies). In conventional IHC/IF, tissue sections are commonly placed on positively charged glass slides (SuperFrost) with polysilane-treated surfaces for improved tissue adhesion, and subsequently covered with thinner glass coverslips to protect tissues during microscopic examination. A special feature of the ChipCytometry technology is the allocation of tissue sections directly on glass coverslips, which are eventually positioned on the back of the microfluidic chips. This raises some difficulties for tissue attachment and structure preservation during usually harsh procedures of antigen retrieval. We therefore compared the performance of two common heat-induced epitope retrieval methods, one procedure with pressure cooker treatment (120°C, 10 min) and another with a more gentle water bath treatment (90°C for 10, 20, or 30 min), in terms of staining quality and tissue preservation on healthy colon tissues. All tested conditions showed comparable staining quality and good signal-to-noise ratio for the selected markers (CD45/CD3 and pan-cytokeratin for, respectively, immune and epithelial cells), despite some variability in staining intensity (Figure 1B, 1C S2A, and S2B). However, we observed huge tissue loss at 120°C in all colon tissue compartments (mucosa, submucosa, and muscularis), compared with much better and robust tissue preservation, in particular for the mucosa, at the sub-boiling temperature of 90°C (Figures 1D and 1E). We identified the 90°C/20 min condition to be the best compromise between tissue integrity and staining intensity for ChipCytometry. Furthermore, we tested pre-treated coverslips, observing an increased tissue integrity in all tested conditions, which therefore allowed us to retrieve FFPE epitopes for ChipCytometry without significant tissue loss (Figures S2C–S2E).

**Figure 2:**
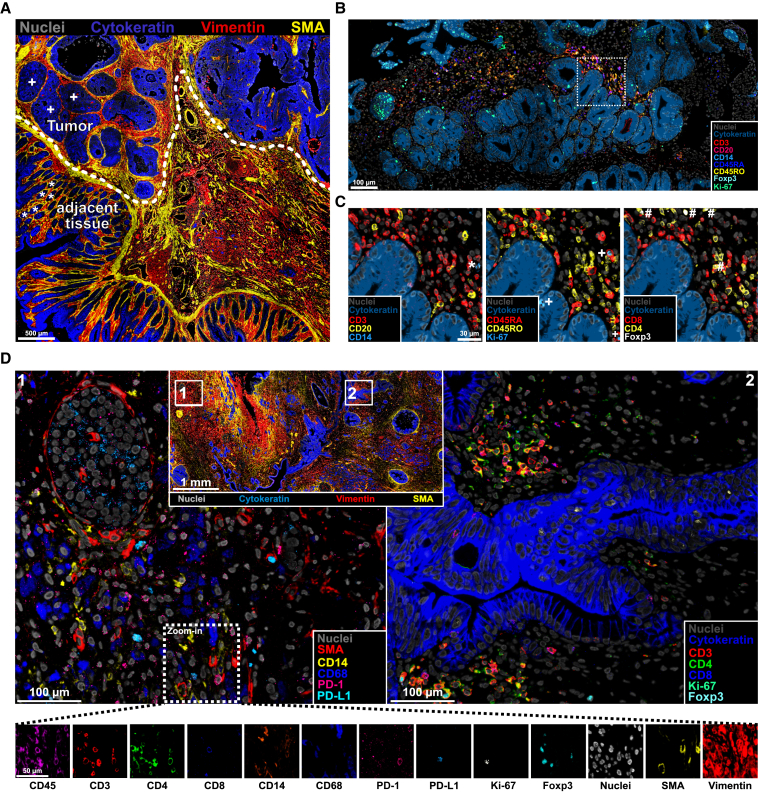
High-quality multiplexed staining of FFPE colon tissues with ChipCytometry (A) Representative image of colon tumor with non-tumor adjacent tissue (above and below the dashed line, respectively). Asterisks (∗) and crosses (+) indicate normal and abnormal crypts, respectively. (B) Representative images of high multiplex staining in an inflamed colon biopsy. Zoom in is indicated by white rectangles and shown in (C). (C) Representative images of mutually exclusive markers (CD3/CD20/CD14, CD45RA/CD45RO, and CD4/CD8) and co-expressed markers (CD4/Foxp3). Exemplary cells are annotated from left to right as ∗, CD14+; +, Ki-67+; #, CD4+Foxp3+ cells. (D) Multiplex ChipCytometry overlay of a human pancreatic cancer tissue (number of markers = 13). See also Figure S4.

To reduce tissue autofluorescence, we tested three of the most established and efficient treatments, namely Sudan Black B, sodium borohydride, and photobleaching (Davis et al., 2014; Yang et al., 2017). We observed Sudan Black B to reduce background autofluorescence the most, with optimal activity achieved with 10 min of incubation (Figures S3A–S3D). No further improvements were obtained by combining Sudan Black B with photobleaching (Figure S3E) or with an alternative autofluorescence treatment using True Black solution (Figure S3F). In addition, we titrated section thickness as this also affects autofluorescence. The best signal-to-noise ratio was obtained with 4 μm thickness, as for higher thicknesses the gain in signal fluorescence was negatively counterbalanced by excessive increase in background autofluorescence (Figure S3G).

### Multiplexed ChipCytometry staining on FFPE tissues

The established sample preparation and staining procedure was applied on tissue sections of various origin (inflamed colon and colorectal, breast, and pancreatic cancer), resulting in staining with very high quality (Figures 2 and S4). Overall, 30 different fluorophore-conjugated antibody clones have been validated so far (Table 1).

**Table 1:** List of antibody clones validated for FFPE tissue staining with ChipCytometry.

Epitope	Conjugate	Filterset	Clone	Species	Company	Catalog number	Dilution	Incubation	Localization
**Fluorophore-conjugated primary antibodies**									

Pan-cytokeratin	AF488	FS488	C11	mouse	BioLegend	628608	1:100	o.n. 4°C	intracellular
CD4	AF488	FS488	polyclonal	Goat	R&D Systems	FAB8165G	1:50	o.n. 4°C	surface
CD14	AF488	FS488	EPR3653	rabbit	Abcam	ab133335	1:100	o.n. 4°C	surface
CD68	FITC	FS488	KP1	mouse	Santa Cruz	sc-20060 FITC	1:100	o.n. 4°C	surface
NF-kB	AF488	FS488	E379	rabbit	Abcam	ab190205	1:50	o.n. 4°C	intranuclear
Vimentin	AF488	FS488	O91D3	mouse	BioLegend	677809	1:300	o.n. 4°C	surface
CD103	AF488	FS488	EPR4166(2)	rabbit	Abcam	ab225152	1:100	o.n. 4°C	surface
Vinculin	AF488	FS488	7F9	mouse	Invitrogen	53-9777-82	1:100	o.n. 4°C	surface
CD45	BUV395	FS395	HI30	mouse	BD Bioscience	563791	1:80	o.n. 4°C	surface
Foxp3	PE	FS560	236A/E7	mouse	eBioscience	12-4777-42	1:30	o.n. 4°C	intranuclear
Ki-67	PE	FS560	B56	mouse	BD Bioscience	556027	1:50	o.n. 4°C	intranuclear
CD45RA	PE	FS560	HI100	mouse	BioLegend	304108	1:600	o.n. 4°C	surface
GATA-3	PE	FS560	L50-823	mouse	BD Pharmingen	560074	1:50	o.n. 4°C	intranuclear
CD8	PE	FS560	C8/144B	mouse	Santa Cruz	sc53212 PE	1:50	o.n. 4°C	surface
CD20	PE	FS560	H1	mouse	BD Bioscience	561174	1:200	o.n. 4°C	surface
CD45RO	PE	FS560	UCHL1	mouse	BioLegend	304206	1:150	o.n. 4°C	surface
PD-L1	PE	FS560	29E.2A3	mouse	BioLegend	329706	1:200	o.n. 4°C	surface
SMA	eF570	FS560	1A4	mouse	eBioscience	41-9760-80	1:500	o.n. 4°C	surface
PD-1	PE	FS560	NAT105	mouse	BioLegend	367404	1:50	o.n. 4°C	surface
pSTAT3	PE	FS560	D3A7	rabbit	Cell Signaling	8119	1:150	o.n. 4°C	intranuclear
Beta-Catenin	PE	FS560	L54 × 10^2^	mouse	Cell Signaling	6898S	1:300	o.n. 4°C	intranuclear
CD133	PE	FS560	clone 7	mouse	BioLegend	372803	1:100	o.n. 4°C	surface
CD79a	PE	FS560	HM47	mouse	BioLegend	333503	1:100	o.n. 4°C	surface
Annexin A1	PE	FS560	EPR19342	rabbit	Abcam	ab225512	1:100	o.n. 4°C	surface
CD57	PE	FS560	HNK-1	mouse	BioLegend	359611	1:100	o.n. 4°C	surface
E-Cadherin	PE	FS560	24E10	rabbit	Cell Signaling	7559S	1:100	o.n. 4°C	surface
MUC2	PE	FS560	SPM296	mouse	Novus	34757PE	1:300	o.n. 4°C	Intracellular
CD123	PE	FS560	6H6	mouse	BioLegend	306006	1:150	o.n. 4°C	surface
CD45	PerCP/Cy5.5	FSPerCP	HI30	mouse	BioLegend	304028	1:50	o.n. 4°C	surface
Ki-67	PerCP/Cy5.5	FSPerCP	B56	mouse	BD Bioscience	561284	1:50	o.n. 4°C	intranuclear
CD20	PerCP/Cy5.5	FSPerCP	H1	mouse	BD Bioscience	558021	1:25	o.n. 4°C	surface
CD45RA	PerCP/Cy5.5	FSPerCP	HI100	mouse	BioLegend	304122	1:100	o.n. 4°C	surface
CD56	PerCP	FSPerCP	123C3.D5	mouse	Novus	33132PCP	1:100	o.n. 4°C	surface
CD45RA	BV421	FS421	HI100	mouse	BioLegend	304129	1:100	o.n. 4°C	surface

**Unconjugated primary antibody**									

CD3	unconjugated	–	SP7	rabbit	Thermo Scientific	RM-9107-S1	1:150	o.n. 4°C	surface

**Fluorophore-conjugated secondary antibodies**									

anti-Rabbit	FITC	FS488	polyclonal	donkey	BioLegend	406403	1:200	2 h RT	secondary
anti-Rabbit	PE	FS560	polyclonal	donkey	BioLegend	406421	1:300	2 h RT	secondary

**Nuclei staining**									

Hoechst	–	FS395	–	mouse	Thermo Scientific	H3570	1:50,000	5 min RT	intranuclear

FITC, fluorescein isothiocyanate; NF-kB;PE, phycoerythrin; nuclear factor kappa-b; o.n., overnight; RT, room temperature.

Pan-cytokeratin, vimentin, and small muscle actin (SMA) markers allowed a broad investigation of the general tissue architecture by discriminating between epithelial, stromal, and muscular cells. As a proof of principle, we stained FFPE sections containing both tumor and non-cancerous adjacent colon tissues. Qualitative analyses of this simple three-marker panel at a low magnification level revealed well-known architectural changes associated with malignant transformation, such as disruption of crypts (Figure 2A).

Due to their heterogeneity, the assessment of immune cells requires a higher multiplexed staining for appropriate phenotyping. For this purpose, we established a large panel of fluorophore-labeled primary antibodies, including, among others, lineage-specific markers such as CD3 (T cells), CD8 (cytotoxic T cells), CD4 (helper T cells), Foxp3 (regulatory T cells [Tregs]), CD20 (B cells), CD14/CD68 (monocytes/macrophages), and CD56 (natural killer cells), and the phenotypic markers CD45RA/CD45RO (naive/effector versus memory cells), PD-1 (activation or exhaustion), and Ki-67 (proliferation) (Figures 2B, S4A, and S4B; Table 1). Absence of overlap between mutually exclusive markers, as well as overlaying of co-expressed markers, demonstrated high specificity of the staining (Figures 2C and 2D).

Finally, markers for epithelial/mesenchymal state, inflammation, stem cells, and mucosa repair mechanisms have been implemented in order to gain more insights into the epithelial compartment (Figures S4C–S4I and Table 1).

### Improving automated quantification of staining intensity by cell-type-specific segmentation and spatial spillover correction

Highly multiplexed imaging generates extremely large amounts of data due to the annotation of a large number of markers to their exact (subcellular) position within a complete tissue section. Manual counting is still an often-used procedure for quantification of cells positive for a single marker, although it is extremely time consuming and error prone (Parkash et al., 2010); thus, manual integration of data from a multitude of markers becomes unrealistic.

**Figure 3:**
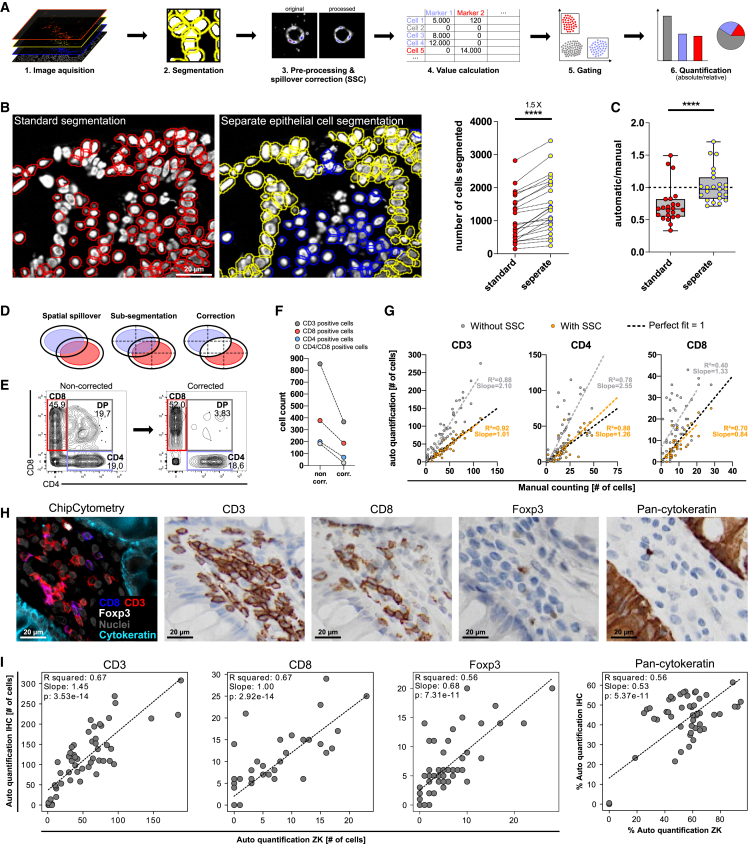
Improved automated analysis of multiplexed imaging data by cell-type segmentation and SSC (A) Schematic representation of the workflow for automated quantification of signal intensities. After image acquisition, cells are segmented, outliers are removed, and the grayscale intensity is measured for each cell and each marker after proper pre-processing and SSC. The resulting [cell × marker] matrix can be further processed to enable quantification of cell subsets by using gating strategy using FlowJo software, similar to analyses of flow cytometry data. (B) Qualitative representation (left) and quantification (right) of segmented cells in case of standard or separate epithelial/non-epithelial cell segmentation on a selected area of colon tissue samples from three different donors. Each dot represents one position (n per donor ≥ 6). (C) Ratio between the number of cells segmented either via standard or separate cell-type segmentation and the number of cells quantified by manual counting from three donors. Manual counting was performed double blinded. Each dot represents one position (n per donor ≥ 6). Data are depicted as interquartile range with whiskers extending from the minimum to the maximum of the dataset. (D) Schematic depiction of SSC. Cells are sub-segmented into quadrants and the signal of a marker is deleted if the fraction of quadrant signal/total signal is exceeding a threshold value. (E) Representative raw data showing CD4 and CD8 staining either with or without SSC. (F) Quantification of cells positive or negative for CD8 and CD4 staining. Signal intensities were automatically quantified with or without SSC and cells were gated according to signal intensity. Cell count refers to the number of cells present in each corresponding gating. (G) Positive cells for CD3, CD4, and CD8 markers were quantified by either manual counting or automatic quantification, with or without SSC. Each dot represents one out of 10 representative positions for three donors. For automated quantification, number of positive cells was obtained by gating strategy using FlowJo software. Correlation analysis was done by Pearson's correlation. (H and I) Human healthy colon sections were stained with antibodies against pan-cytokeratin, CD3, CD8, and Foxp3. (H) Representative images of multiparameter ChipCytometry and individual marker IHC on consecutive slides. (I) Pearson's correlation between automated quantification of ChipCytometry staining and automated quantification of IHC staining (using a commercially available software). Each dot represents one out of 15 positions analyzed per donor (n = 3). In (B) and (D), statistical testing was conducted by paired t test (∗∗∗∗p < 0.0001). See also Figures S5 and S6)

We therefore adapted a software tool developed by Lin et al. for automated quantification of IF signal intensities (Lin et al., 2015, 2018), as the crucial initial step for further computational analyses of high-dimensional data. In the method described by Lin et al., after imaging acquisition, cells are first segmented via nuclei staining, the obtained regions of interest are enlarged to encompass membranes, and finally intensity values are measured for all acquired markers (Figures 3A and Figure S5A–5C). The quantified signal intensity data can be eventually converted in formats useful for several analyses (e.g., unsupervised clustering, heatmaps, or neighborhood analyses). In this article, quantified data have been mainly analyzed with software for flow cytometry data; as with flow cytometry staining of single-cell suspensions, manual gating was used to identify and quantify tissue-derived single cells positive for one or more markers (Figure 3A).

We implemented three main steps in the workflow described above, which resulted in more accurate quantifications of cells specific for one or more markers. First, we applied an independent segmentation of epithelial and non-epithelial cells in healthy colon tissues, which yielded a 1.5-fold increase in the total number of segmented cells on average (Figures 3B and S5D). We further observed a similar number of segmented cells between our cell-type-specific segmentation and manually counted nuclei, compared with the conventional segmentation approach (Figure 3C). Overall, less information is lost due to underestimation of cells in a tissue. This approach of separate segmentation was broadly tested on several human tissue types, achieving comparable results (Figure S5E). Secondly, after segmentation, surface marker signals were pre-processed to reduce noise (for example, from antibody aggregates) and achieve a sharp signal with a low probability of spillover to neighboring cells.

As a last step, we developed an approach for removing false-positive signals coming from spatial spillover. In tissues with high cell densities, the likelihood is higher that a signal of an individual cell physically extends into neighboring cells. In addition, unspecific binding of antibody aggregates could occur. With spatial spillover correction (SSC), only signals covering the majority of a cell surface are accepted (Figure 3D). As proof of principle, we applied SSC to staining of CD4 and CD8 surface markers, known to be mutually exclusive on the vast majority of mature T cells, and observed a drastic reduction of double-positive cells after correction (Figures 3E, S6A, and S6B). Automated quantification of CD8/CD4 single-positive, double-negative (DN), and double-positive (DP) cells confirmed the substantial drop of DP+ cells when SSC was applied but also revealed a significant reduction in absolute counts for the other three populations (Figure 3F), highlighting how much spatial spillover can interfere with appropriate signal quantifications.

To evaluate in more detail the effect of SSC on accuracy of signal quantifications, we compared our method (either with or without SSC) with manual counting. The number of cells defined as positive for CD3, CD4, or CD8 by using uncorrected automated quantification positively correlated with the manual counting, but with an overestimation in quantification. SSC not only further improved the existing correlations with manually counted cells but also eliminated the bias of higher numbers of cells assigned positive for the analyzed markers. Remarkably, SSC quantification closely matched manual quantification, indicating that SSC eliminated almost all false-positive signals without biasing the number of true-positive cells (Figure 3G). Best correction performance was observed with a threshold of 60% (cells are excluded if signal of a cell quadrant is higher than 60% of the total cell signal) (Figures S6C–S6E).

To validate the robustness of the developed automated quantification, we compared our approach with IHC, still the gold-standard method for marker staining in diagnostics. Consecutive slides were stained either using IHC for individual markers (CD3, CD8, Foxp3, and pan-cytokeratin) or ChipCytometry for the four markers simultaneously (Figure 3H). Interestingly, automated quantification of ChipCytometry staining robustly correlated with quantification of IHC staining for all the evaluated markers (Figure 3I). The use of consecutive slides accounted for most of the variability observed between ChipCytometry and IHC quantifications. Indeed, improved correlation was observed when the same tissue section was used for both IHC and ChipCytometry staining (Figures S6F and S6G).

### SSC improves the quality and accuracy of clustering analyses

To gain deep insights into tissue biology, simultaneous analysis of a multitude of markers is necessary. For this reason, unsupervised clustering analyses have been applied on quantified staining intensities from HMTI to reveal tissue composition and heterogeneity (Chevrier et al., 2017; Lin et al., 2018; Wagner et al., 2019). In light of the effect of spatial spillover on the accuracy of staining quantification, we evaluated whether and to what extent it could affect dimensional reduction and clustering of high-dimensional data.

**Figure 4:**
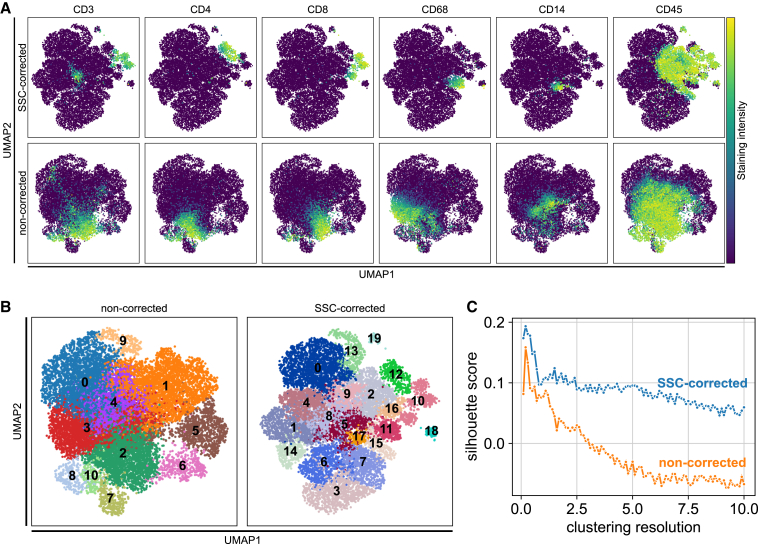
SSC improves quality of clustering analyses (A–C) Section from FFPE inflamed gut tissue was stained with 18 markers (CD45, CD45RA, CD45RO, CD3, CD4, CD8, CD14, CD68, CD20, CD25, Foxp3, Gata3, pan-cytokeratin, Ki-67, PD-1, PD-L1, vimentin, SMA). Protein expression, by means of staining fluorescence intensity, was used as input to perform neighborhood embedding. (A) UMAP neighborhood embeddings showing distribution of CD8, CD4, CD3, CD68, CD14, and CD45 markers in presence or not of SSC. (B) Depiction of Leiden clustering (resolution = 0.3) according to the neighborhood embedding. Each number represents one cluster. (C) Silhouette score for the Leiden clustering was calculated based on the UMAP coordinates for clustering resolutions ranging from 0 to 10 in steps of 0.1.

We therefore stained an inflamed colon biopsy with an 18-plex panel, quantified signal intensities either with or without application of SSC, and performed embedding based on protein expression via uniform manifold approximation and projection (UMAP) (Becht et al., 2019). As representative examples, we analyzed the distribution of CD3, CD8, CD4, CD68, CD14, and CD45 markers that should define cytotoxic T cells (CD45+CD3+CD8+), helper T cells (CD45+CD3+CD4+), macrophages (CD45+CD68+), and monocytes (CD45+CD14+). Broader expression of individual markers was observed in UMAPs generated from non-SSC-quantified data compared with SSC-quantified data (Figure 4A). This resulted in a higher degree of co-expression of markers supposed to be mutually exclusive if no SSC was applied; for example, coincidence of CD68 with CD3 and CD8 with CD4. In contrast, such co-expression artifacts were absent in UMAPs of SSC-corrected data (Figure 4A).

The quality of signal quantification is reflected in the quality of clustering. Without correction of spatial spillover, Leiden clustering (Traag et al., 2019) of the neighborhood graph revealed a total of 10 clusters, with some faint clusters mainly obscured by and overlapping with other clusters (e.g.., cluster 4 and 3). In contrast, a higher number of more defined clusters was obtained from SSC data (Figure 4B), using the same parameters for embedding and clustering resolution. Remarkably, CD3+CD8+ and CD3+CD4+ cells could not be clustered independently if SSC was not applied (cluster 2 in non-SSC UMAPs versus clusters 10 and 12 in SSC UMAPs). Similarly, CD14+ monocytes were not distinctly clustered in non-SSC UMAPs (cluster 3 and 4) but formed a well-defined clustered in SSC UMAPs (cluster 17) (Figure 4B). The benefit in terms of cluster quality provided by SSC was confirmed by consistent high silhouette scores despite increasing clustering resolution (Figure 4C).

### Identification of phenotypically complex rare cells by gating strategy on automated quantified multiplexed imaging

Unsupervised clustering analyses showed success in capturing tissue complexity and changes in pathological conditions (Lin et al., 2018; Wagner et al., 2019). However, a major drawback is that information about rare but biologically relevant populations may be overlooked in such global analysis of HMTI data.

One example of such a population is Tregs. Tregs play a crucial role in maintaining immune tolerance (Sakaguchi et al., 2008), and their skewed reconstitution after hematopoietic stem cell transplantation has been associated with graft-versus-host disease (GvHD) (Edinger et al., 2003; Taylor et al., 2002). Therefore *in situ* analyses of Tregs is relevant for both research and diagnostic purposes. However, their low frequency and phenotypic complexity (Tanoue et al., 2016) complicate their evaluation in human tissues, in particular in samples of limited size.

**Figure 5:**
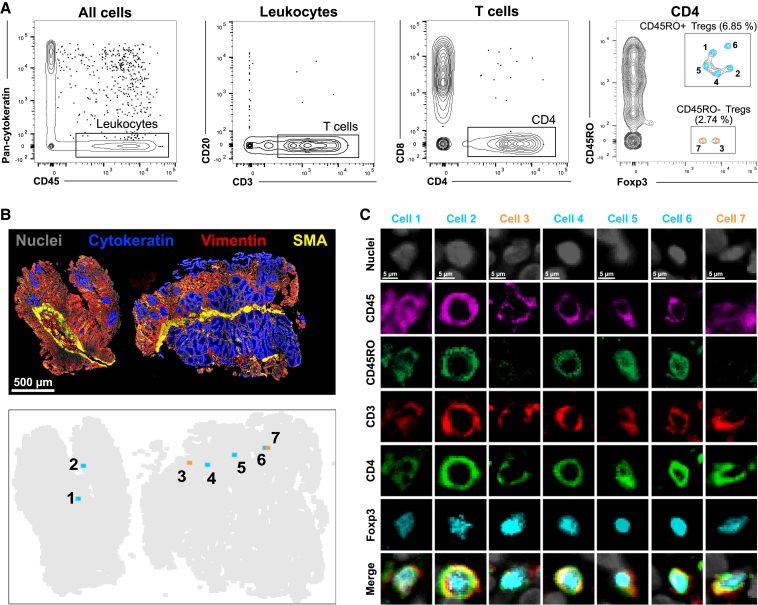
Detection of rare phenotypically complex cells using gating strategy on automated quantified signal intensities (A–C) Tissue section from GvHD gut biopsy was stained with 18 markers (CD45, CD45RA, CD45RO, CD3, CD4, CD8, CD14, CD68, CD20, CD25, Foxp3, Gata3, pan-cytokeratin, Ki-67, PD-1, PD-L1, vimentin, SMA). (A) Representative depiction of gating strategy used to navigate into tissue composition. From all segmented cells, Tregs were identified by sequential gating according to CD45+CD3+CD4+Foxp3+ expression and finally discriminated according to CD45RO expression. (B) Example of replotting of a gated population into the original stained tissue, to assess its spatial distribution/location. (C) Original staining images of the seven Tregs identified in (B).

We attempted to identify Tregs in size-limited gut biopsies from GvHD patients. To assess this population, we used a gating strategy similar to that of flow cytometry data analyses, identifying Tregs by sequential gating according to CD45, CD3, CD4, and Foxp3 expression (Figure 5A). Additionally, we phenotyped Tregs according to CD45RO expression, which discriminates activated versus resting Tregs (Miyara et al., 2009). Seven cells were identified in total and the low frequency of these phenotypically complex cells would have made a visual identification tedious and prone to errors. Importantly, the spatial information of Tregs was preserved as gated cells could be replotted and reconciled with their original position within the tissue (Figure 5B). Detailed evaluation of the staining quality of individual markers at single-cell level confirmed the reliability of the identified cells (Figure 5C).

## Discussion

The work presented here describes for the first time the successful transfer of highly multiplexed imaging to ChipCytometry on routine FFPE tissue sections. The high staining quality and single-cell resolution of this approach allowed us to apply software solutions (available as open source) for automated quantification of signal intensities. Remarkably, we implemented an SSC to remove artificial signals from staining on neighboring cells, which resulted in higher accuracy of cell-type quantifications and phenotyping.

Besides common problems intrinsic to optical imaging of FFPE tissues (i.e., antigen retrieval and autofluorescence; Shi et al., 2011; Viegas et al., 2007), a major challenge posed by ChipCytometry is the use of thin glass coverslips for mounting tissue sections, which could compromise tissue adherence. Here we show that the use of milder antigen retrieval conditions can largely avoid this problem while maintaining excellent antigen accessibility. Furthermore, the availability of commercially produced surface-treated coverslips, similar to Superfrost glass slides, will be an advantage with regard to this challenge in the future.

Overall, we provide an optimized protocol for sample preparation and antigen retrieval, which enabled high-quality staining in a variety of epithelial tissues (colon, breast, and pancreas) and could represent a robust starting point for applying ChipCytometry HMTI in other tissue types. We established a panel of 30 markers with a specific focus on immune cells, as clinically, the quantification of immune cells in solid tumor tissue sections has gained tremendous importance for immune-checkpoint therapy evaluation and prognosis (Angell et al., 2020). Increasing in-depth phenotype profiling would eventually facilitate design of individualized therapeutic approaches. Additionally, we validated markers useful for analyzing the general tissue architecture (pan-cytokeratin, E-cadherin, vimentin, vinculin, and SMA) and gaining insight into the epithelial compartment (epithelial/mesenchymal, inflammation, and stem cell markers). The number of directly conjugated antibodies that are commercially available for FFPE tissue staining is likely to significantly increase over the coming years, thereby reducing the efforts in antibody clone searching and enhancing the information that can be retrieved using ChipCytometry.

Concerning automated quantification, its efficacy is challenged by the different types, and thus shapes, of cells composing a tissue, and at times by high cellular density (Vu et al., 2019). These, respectively, can limit the yield of cell segmentation and generate staining artifacts due to signal spillover from neighboring cells. For cell segmentation, we started from common algorithms for thresholding and watershed (Lin et al., 2015) but importantly, we implemented a cell-type-specific segmentation pipeline that resolved up to 90% of all nucleated cells. This segmentation approach was tested on several tissue types, for which optimized parameters for separate segmentation are available in our repository, and can be easily expanded to additional cell types. In addition, staining artifacts were efficiently removed by applying first a fine-tuned image processing before value calculation, which produced a sharp grayscale signal and reduced background, followed by an SSC. We showed that staining artifacts due to spatial spillover not only alter the count of cells assigned to one or more markers but also bias cell phenotypes and thus the interpretation of tissue compositions. This problem was efficiently tackled for tissue mass cytometry (Chevrier et al., 2018). Our approach for SSC showed consistent minimization of staining artifacts for optical imaging, which in addition to ChipCytometry data, can be applied to any HMTI data.

Altogether, the image processing pipeline implemented in this study, which is available open source, allows users to automatically move from exported images to quantified fluorescence intensities. Furthermore, the pipeline can be applied to all imaging data regardless of the acquisition method, provided grayscale images for each channel are available (or data can be converted into this format). Preliminary analysis of third-party imaging data (confocal microscopy, slide scanner data) has been performed and integrated in our online open repository.

The matrix of quantified staining intensities can be used for many purposes. Initial interest focused on unraveling tissue composition and heterogeneity (Chevrier et al., 2017; Lin et al., 2018; Wagner et al., 2019), as the large quantities of information generated by HMTI allowed clustering analyses according to protein expression simply by applying existing software solutions developed for single-cell RNA sequencing (i.e., Scanpy; Wolf et al., 2018). Although not the principal focus of this article, we have shown here that these clustering analyses can be usefully applied to ChipCytometry HMTI (Figure 4). Despite being highly relevant with broad applications to pre-clinical research, these approaches may be less applied in clinical histopathology. In this context, the use of HMTI for the parallel quantification of different cell types or phenotypically complex populations could be easier to implement. In this article, we used Tregs in gut GvHD biopsies as proof of principle for the *in situ* detection and quantification of rare and phenotypically complex populations. Appropriate Treg phenotyping requires the analyses of many markers as well as intracellular Foxp3, the most commonly used marker for Treg, which can also be transiently expressed by cell populations other than CD4 T cells (Devaud et al., 2014; Morgan et al., 2005). We showed that Tregs could be easily detected and counted by applying gating strategies for marker expression to data of quantified staining intensities, a typical approach for flow cytometry analysis.

In summary, we have demonstrated here that HMTI and quantified data analyses are now possible with ChipCytometry. This, combined with unique features of the platform itself (such as HDR of image acquisition, tissue biobanking, and possibility of automated processing), makes ChipCytometry a suitable technique for broad application both in routine research and clinical pathology.

### Limitations of the study

As with all technologies, ChipCytometry also has some limitations that require further improvements and optimizations. Directly conjugated primary antibodies for FFPE tissues are not as broadly available as for fresh-frozen samples, therefore an extensive search for working clones is needed to improve multiplexing. Furthermore, the acquisition of highly multiplexed staining is still of intermediate throughput, as only up to five markers can be stained simultaneously (a 30-plex, for example, would require approximately 2 weeks); the application of the CODEX staining principle to ChipCytometry could be envisioned as an alternative to improve throughput by the co-staining of more markers. Photobleaching may also be a time-consuming step, in particular if large samples are analyzed. We here provided proof of principles on the feasibility of faster chemical bleaching on ChipCytometry. Finally, we observed two limitations in steps of imaging and data processing. Firstly, the use of photobleachable dyes led to imaging artifacts at the edges of individual positions, which are the result of a double exposure of such areas to light during imaging and photobleaching. Eventually, areas with decreased fluorescent signals are generated. Secondly, common to all other HMTI techniques and associated methods for staining quantification, the workflow provided here suffers from steps of manual normalization and thresholding, despite careful titration. Machine-learning approaches for automated cell segmentation that are also capable of quantifying signal intensities directly from tissue imaging are highly needed and will move the field toward even more objective analyses. To overcome these limitations, a software option for removing shading artifacts and a pre-trained neuronal network model for machine-learning-based quantification have been implemented in our script, although more extensive testing is still necessary to validate this.

## STAR★Methods

### Key resources table

**Table:** 

REAGENT or RESOURCE	SOURCE	IDENTIFIER
**Antibodies**		

Anti-human pan-cytokeratin (AF488)	BioLegend	RRID: AB_2616664
Anti-human CD4 (AF488)	R&D systems	RRID: AB_2728839
Anti-human CD14 (AF488)	Abcam	RRID: AB_2889158
Anti-human CD68 (FITC)	Santa Cruz	Cat#: sc-20060 FITC
Anti-human NF-kB (AF488)	abcam	Cat#: ab190205
Anti-human vimentin (AF488)	BioLegend	RRID: AB_2650955
Anti-human CD103 (AF488)	abcam	RRID: AB_2884944
Anti-human vinculin (AF488)	Thermo Fisher Scientific	RRID: AB_2574473
Anti-human CD45 (BUV395)	BD Biosciences	RRID: AB_2744400
Anti-human Foxp3 (PE)	Thermo Fisher Scientific	RRID: AB_1944444
Anti-human Ki-67 (PE)	BD Biosciences	RRID: AB_2266296
Anti-human CD45RA (PE)	BioLegend	RRID: AB_314412
Anti-human GATA-3 (PE)	BD Biosciences	RRID: AB_1645330
Anti-human CD8 (PE)	Santa Cruz	RRID: AB_1120718
Anti-human CD20 (PE)	BD Biosciences	RRID: AB_10563904
Anti-human CD45RO (PE)	BioLegend	RRID: AB_314422
Anti-human PD-L1 (PE)	BioLegend	RRID: AB_940368
Anti-human SMA (eFluor570)	Thermo Fisher Scientific	RRID: AB_2573630
Anti-human PD-1 (PE)	BioLegend	RRID: AB_2566065
Anti-human pSTAT3 (PE)	Cell Signaling Technology	RRID: AB_10859889
Anti-human beta-Catenin (PE)	Cell Signaling Technology	RRID: AB_10828097
Anti-human CD133 (PE)	Biolegend	RRID: AB_2632879
Anti-human CD79a (PE)	Biolegend	RRID: AB_1089076
Anti-human Annexin A1 (PE)	abcam	Cat#: ab225512
Anti-human CD57 (PE)	Biolegend	RRID: AB_2562758
Anti-human E-Cadherin (PE)	Cell Signaling	RRID: AB_10950323
Anti-human MUC2 (PE)	Novus	Cat#: 34757PE
Anti-human CD123 (PE)	BioLegend	RRID: AB_314580
Anti-human CD45 (PerCP/Cy5.5)	BioLegend	RRID: AB_893338
Anti-human Ki-67 (PerCP/Cy5.5)	BD Bioscience	RRID: AB_10611574
Anti-human CD20 (PerCP/Cy5.5)	BD Bioscience	RRID: AB_396990
Anti-human CD45RA (PerCP/Cy5.5)	BioLegend	RRID: AB_893357
Anti-human CD56 (PerCP)	Novus	Cat#: 33132PCP
Anti-human CD45RA (BV421)	BioLegend	RRID: AB_10900421
Anti-human CD3	Thermo Fisher Scientific	RRID: AB_149924
Anti-rabbit IgG	Biolegend	RRID: AB_893531
Anti-rabbit IgG	Biolegend	RRID: AB_2563484

**Biological samples**		

Healthy adjacent tissues from colon resections	Molecular Tumor Biology (Prof. Dr. Klaus-Peter Janssen) at the Dept. of Surgery (TUM)	N/A
Biopsies from aHSCT patients	University hospital of Regensburg (Prof. Ernst Holler)	N/A
Colorectal cancer resections	TUM Pathology Department (Dr. Katja Steiger)	N/A
Pancreatic cancer resections	TUM Pathology Department (Dr. Katja Steiger)	N/A
Breast cancer resections	TUM Pathology Department (Dr. Katja Steiger)	N/A

**Chemicals, peptides, and recombinant proteins**		

Hoechst	Thermo Fisher Scientific	Cat#: H3570
Ethanol absolute, 1 % MEK	Carl Roth	Cat#: K928
Ethanol 70%, 1 % MEK	Carl Roth	Cat#: T913
Roticlear	Carl Roth	Cat#: A538
Tween-20	Carl Roth	Cat#: 9127
Tris(hydroxymethyl)aminomethane (TRIS)	Carl Roth	Cat#: 9429
Ethylenediaminetetraacetic acid (EDTA)	Carl Roth	Cat#: X986
Sudan Back B	Sigma Aldrich	Cat#: 199664
True Black	Biotium	Cat#: 23007
Dulbecco's phosphate-buffered saline (DPBS)	PAN-Biotech	Cat#: P04-36050P
Sodium Borohydride	Sigma Aldrich	Cat#: 71320
Hydrogen peroxide (H_2_O_2_)	Sigma Aldrich	Cat#: H1009

**Deposited data**		

Code and Dataset for pipeline testing	This publication	https://github.com/SebastianJarosch/ChipCytometry-Image-Processing

**Software and algorithms**		

ImageJ 1.53c	Schindelin et al., 2012	https://imagej.net/software/fiji/
Affinity photo V1.8.3	Serif Europe Ltd. 2020	https://affinity.serif.com/
FlowJo 10	FlowJo LLC	https://www.flowjo.com
Prism 9	Graphpad	https://www.graphpad.com
Aperio ImageScope 12.4	Leica Biosystems	https://www.leicabiosystems.com/de/digitalpathologie/verwaltung/aperio-imagescope/
Scanpy 1.8	Wolf et al. (2018)	https://github.com/theislab/scanpy
Matlab R2018b	Mathworks	https://de.mathworks.com/products/matlab.html
Automated fluorescence signal quantification	This study	Zenodo https://doi.org/10.5281/zenodo.5533411

### Resource availability

#### Lead contact

Further information and requests for resources and reagents should be directed to and will be fulfilled by the lead contact, Dirk H. Busch (dirk.busch@tum.de).

#### Materials availability

This study did not generate new unique reagents.

### Experimental model and subject details

#### FFPE human samples

Healthy tissue samples (Table S1, Samples 1-3) were kindly provided by the group of Molecular Tumor Biology (Prof. Dr. Klaus-Peter Janssen) at the Dept. of Surgery (TUM) and were derived from non-diseased tissue from surgical colectomy of colorectal cancer patients. Inflamed colon tissue biopsies (Table S1, Samples 4+5) were kindly provided by Prof. Dr. Ernst Holler from patients who experienced GvHD after HSCT enrolled at the University hospital of Regensburg. Cancer tissues (pancreatic, colon, and breast) were kindly provided by Dr. Katja Steiger from TUM Pathology Department (Table S1 Samples 6-16). Information about age, sex, and disease status is available in Table S1. All procedures were approved by local ethics committee (ethical committee of the School of Medicine, Technical University Munich - 330/18S; ethical committee of the University of Regensburg - 09/059 and 18-684482-101; ethical committee of the Klinikum Rechts der Isar, Technical University Munich - 322/18 S-AS) and after informed, written consent of patients regarding use of the tissue samples.

### Method details

#### Preparation and staining of tissue sections

##### Preparation of FFPE tissues

Tissue sections (4-5 μm) were mounted on glass coverslips (24 x 50 mm, 1 mm thickness, Engelbrecht Automat-Star #K12460A1,0) and dried for at least 10 h before the paraffin melting overnight at 60°C. To completely melt, tissues were heated at 70°C for additionally 30 min and immediately immersed in Xylene for 10 min. The Xylene washing was repeated in two other Xylene-containing dishes for 10 min, followed by 2 x 10 min absolute ethanol incubation. Rehydration was achieved by sequentially immersing slides in staining dishes containing 90% ethanol, 70% ethanol, 50% ethanol and tap water for 5 min each. The antigen retrieval was performed in a water bath containing a dish with a basic retrieval buffer (10 mM TRIS, 1 mM EDTA, pH 8.5) at 90°C for 20 min. The coverslips were transferred into PBS at RT to let them cool down before loading onto the chip, according to manufacturer's recommendations. Chips were rinsed with PBST before sections were blocked in 500 μl goat serum (5% in PBST) for 1 h at RT. For decreasing autofluorescence, chips were rinsed with 500 μl Sudan Black B solution (0.1% in 70% EtOH), incubated for 10 min and extensively washed with 70% EtOH and PBST (0.1% Tween-20 in PBS). A first background was acquired in the FITC channel to locate the tissue on the chip and select the desired positions.

For antigen retrieval testing, slides were heated in a staining dish containing the retrieval buffer (10 mM TRIS, 1 mM EDTA, pH 8.5) for 10, 20 or 30 min using a water bath at a buffer temperature of 90°C or directly in a pressure cooker for 10 min at ∼120°C.

For autofluorescence treatment tests, Sudan Black B, True Black, sodium borohydride and photobleaching were tested. For Sudan Black B treatment, sections have been incubated for 5, 10, 20 or 30 min in a 0.1% (m/v) solution of Sudan Black B in 70% EtOH before they were washed extensively with first 70% EtOH and subsequently PBST. For True Black treatment, sections were incubated for 5 min at RT with 5% (v/v) solution of True Black in 70% EtOH before they were washed extensively with first 70% EtOH and subsequently PBST. For sodium borohydride, sections were incubated 3 x 10 min in 0.1% (m/v) solution in PBS. Sections were washed extensively with PBST. Photobleaching was achieved by incubating the sections in PBST under a white light source for 30 min and the buffer was exchanged every 5 min to minimize heating of the section during this procedure.

For testing of tissue adherence, tissue sections were mounted on either glass coverslips, polysilane-coated microscopy slides (Thermo scientific Superfrost® Plus #J1800AMNZ) or pre-treated coverslips (kindly provided by Zellkraftwerk company).

##### ChipCytometry staining, imaging, and bleaching

The platform used in this study is the Zellscanner ONE, an upright inverted microscope (Zeiss Axio Observer 7) mainly equipped with i) a lamp for fluorophore excitation (Zeiss HBO 100 Microscope Illuminator), ii) a pre-defined set of filters to simultaneously detect five different fluorescent dyes (BUV395, BV421, FITC, PE and PerCP-Cy5.5; Table S2) and a iii) motorized scanning table. The high dynamic range (HDR) acquisition of images with a CCD camera (Basler scA1400-17gm, 1392 x 1040 pixel, 6.45 μm x 6.45 μm pixel size) allows true quantification of up to 4.2 billion grayscale intensities (32 bit HDR by exposure fusion). For staining, cocktails of fluorophore-conjugated primary antibodies were centrifuged for 10 min at 14.000 rpm before loading into the chip to avoid transfer of dye aggregates. Before antibody incubation, tissue-containing chips were positioned under the ChipCytometry microscope to acquire background autofluorescence for each channel used in the staining, which was eventually subtracted from final images. Chips were then manually rinsed with PBST and incubated with 300 μl of the antibody cocktail overnight at 4°C, followed by extensive washing with 15 ml PBST. Primary antibodies could also be incubated for shorter time or at RT after proper titration. For CD3, fluorophore-conjugated secondary antibody (1:300) was incubated for 2 h at RT. Hoechst 33342 (1:50000) was incubated for 5 min at RT prior to acquisition. Finally, chips were re-positioned under the microscope and images acquired according to the following exposure times: 300 ms for PE, 500 ms for FITC, 300 ms for PerCP-Cy5.5, 50 ms for BV421 and 1000 ms for BUV395. HDR images are automatically generated by gradually decreasing the sensitivity of the detector camera for each acquisition. Images were acquired with a Zellscanner One Chip cytometer (Zellkraftwerk) using a 20x objective (Zeiss Plan-APOCHROMAT 20x/0.8) and the dedicated ZellExplorer software.

After imaging, photobleaching was performed with the ChipCytometry microscope by exposing each position of the tissue for 20 seconds to white light from the build-in HBO lamp with a 364 nm longpass filter to protect epitope damage from UV light. As alternative, chemical bleaching was performed incubating the chip with fluorophore inactivation buffer (PBS, 24 mM NaOH, 4,5 % H_2_O_2_ as previously described by Lin et al.) for 30 min in the presence of light. In order to avoid drying of the section due to air-bubbles that arise during the oxidative reaction, the buffer was exchanged every 5 min.

#### Data processing

Acquired images were manually adjusted for contrast and background, and exported as grayscale.tiff images. In this format, images can be processed from the ImageJ macro script for automated image processing, which includes all steps from image stitching to fluorescence mean intensity calculation.

##### Generation of images

Contrast and background were adjusted for each marker individually on the background-subtracted HDR images in the ZellExplorer software, and images (one per position) were exported as 16 bit grayscale.tiff for subsequent analysis with ImageJ (V1.53c). Images were stitched by the grid/collection stitching plugin. For visualization of multiple stainings, marker-overlays were prepared using Affinity photo (V1.8.3) by overlaying the stitched grayscale images and applying a gradient in the desired color. The layers with the individual markers were then added to a composite image representing the sum of pixel-intensities in each color channel.

##### Segmentation

Nuclei images have been pre-processed with a Gaussian blurring (convolution with a Gaussian function for smoothing) with a sigma (intensity of blurring) of 1, before the thresholding and converting to a binary image. The threshold therefore was manually adjusted in case the nuclei staining did not fit the predefined parameters. The watershed algorithm on the binary image allows - together with an increased lower threshold - to separate overlapping cells and was followed by the particle detection in ImageJ (epithelial cells: size = 75 - 2000 pixel, minimal circularity = 0.2; non-epithelial cells: size = 70 - 400 pixel, minimal circularity = 0.55). The resulting regions of interest (ROIs) were enlarged by 3 pixels to ensure the full coverage of the surface signal. Epithelial and non-epithelial cells were segmented separately by subtracting segmented crypts. Automatic Yen thresholding allowed the segmentation of pan-cytokeratin positive areas with a size of 1000-infinity pixels. These segmented areas were then removed from the nuclei staining channel in order to generate the template for non-epithelial cell segmentation. In a second step, the image of these non-epithelial cells was again subtracted from the original nuclei staining, generating an image for segmentation of only epithelial cells.

##### Image pre-processing

For surface markers, an outlier filter was applied to remove staining artefacts, which replaced a pixel by the median of the pixels in the surrounding if it deviates from the median by more than a threshold value. In addition, a minimum filter (grayscale erosion by replacing each pixel in the image with the smallest pixel value in that pixel neighborhood) was used to reduce the blurring of bright signals towards neighboring cells.

##### Spatial spillover correction

Each ROI was subdivided into four quadrants and the mean intensity for each quadrant as well as for the whole cell was measured. If the factor quadrant intensity/total intensity reached a threshold for one of the sub-ROIs, the signal for the whole cell was deleted and the ROI saved separately to allow investigation of the excluded cells. Spillover correction was only performed on cells with an intensity value greater than 100 to optimize computing time.

##### Fluorescence mean intensity calculation

Mean intensity, X/Y coordinates, area, and circularity were measured afterwards for each cell on each of the pre-processed and spillover-corrected images. The resulting table consists of one row for each cell in each marker and one column for each parameter.

##### Conversion into .fcs files

The intensity value table was converted using MATLAB (V9.5) in a [cells x parameters] matrix, where each row represents a cell and each column one parameter (fluorescence intensity or feature like position, area or circularity). The code for this reformatting step was adaptet from Lin et al. The data matrix can be converted using the *writeFCS (fname, DATA, TEXT, OTHER)* function (Nedbal, 2021) in MATLAB and the resulting file can be analyzed in FlowJo (V10).

#### Data analyses

##### Signal-to-Noise ratio evaluation

Analysis was done on two areas of a tissue, each covering 5 positions. Contrast and background of the HDR image were adjusted (same parameters for each condition) and images exported as 16 bit grayscale.tiff for subsequent analysis with ImageJ. For pan-cytokeratin staining (not bleachable fluorophore), crypts and background regions were segmented as freehand selections in ImageJ and the mean intensity value was measured. The signal-to-noise ratio (SNR) was calculated as crypt intensity divided by the mean of all background intensities. For other markers (bleachable fluorophores), the tissue was bleached after acquisition and a background recorded. The surface of cells was selected as freehand line (3-pixel thickness) and the intensity value measured for both staining and background images. The SNR is defined as the staining intensity divided by the background intensity for each cell.

##### Tissue integrity scoring

Slides or coverslips were incubated in Mayer's Hematoxylin solution for 6 min, immersed in tap water and subsequently incubated in Eosin (1%) for 6 min. After washing in tap water, slides were incubated in 70% ethanol for 1 min, followed by 2 x 5 min absolute ethanol and 3 x 5 min Xylene incubation for dehydration. Mounting with either a coverslip or a slide was followed by drying over night before the sections were scanned with a slide scanner (Olympus BX61VS, 20X). Integrity was evaluated by a trained pathologist from 0 (no tissue loss) to 3 (complete loss of tissue).

##### Manual counting

Cells or positive staining events were counted blinded by two scientists and the mean of the counts was used for plotting and further analysis. Only complete cells with an ordinary shape and complete surface marker stainings were taken into account for counting.

##### Computer-assisted image analysis of IHC stainings

The IHC staining was performed on the Ventana Benchmark XT from Roche with the Ultra View Universal DAB Kit using a rabbit antibody against CD3 and mouse antibodies against CD8, pan-cytokeratin and Foxp3. Briefly, the slides were deparaffinized using deparaffinization solution. For pan-cytokeratin staining, the antigen retrieval was performed by pre-treating the tissue with Protease1 (from Roche) for 8 min while for other stainings, CC1 (Cell Conditioning 1 from Roche) was used for 60 min. All antibodies were incubated for 32 min Counterstaining was done with hematoxylin. Slides were scanned in an Aperio AT2 slide scanner (Leica) at magnification of 40X. The immunohistochemical expression was analyzed with Aperio ImageScope software (V12.4.0.7018). “Positive Pixel Count v9” algorithm was used for quantifying pan-cytokeratin expression. The total positive pixel was normalized to the total area of the annotated regions on tissue section (pixel/mm2). “Nuclear v9” algorithm was used for CD3 and CD8 positive cell count. The default set of parameters of the algorithms was modified according to the stain contrast and intensity of the scanned images.

##### Embedding of multiplexed imaging data

The SCANPY package (V1.8.0) developed for the analysis of scRNA sequencing data in python (Wolf et al.) was used to run clustering analysis of the imaging data. The value table generated in ImageJ was therefore re-formatted to a [cells x parameters] matrix as described above with MATLab to be able to import the intensity values per cell as adata object. Cells with less than three proteins expressed and unusually large cells were excluded. Furthermore, the whole data matrix was normalized, logarithmized, and scaled for further processing. A principal component analysis (PCA) was followed by construction of the neighborhood graph for the 50 nearest neighbors and the first seven principle components with the UMAP (Becht et al.) method and a Euclidian metric. Clustering was performed using the Leiden algorithm (Traag et al.) with a titrated resolution of 0.3.

### Quantification and statistical analysis

Data were displayed using Graphad Prism (V9.1) or the seaborn (0.10.0) and matplotlib (3.1.3) packages in python. Statistical and correlation analyses were performed in Prism and with stat.linregress method from the scipy (1.4.1) module, respectively. Significance is defined as ∗p-value < 0.05, ∗∗p-value < 0.01,∗∗∗p-value < 0.001, ∗∗∗∗p-value < 0.0001. Information on statistical tests used for individual figures can be found in the figure legends.

## Data Availability

• Example data to test the pipeline described in this paper is available at https://github.com/SebastianJarosch/ChipCytometry-Image-Processing. • All original code has been deposited at GitHub and is publicly available as of the date of publication. The DOI (https://doi.org/10.5281/zenodo.5533411) is listed in the key resources table. • Any additional information required to reanalyze the data reported in this paper is available from the lead contact upon request.

## Supplemental information

Document S1. Figures S1–S6 and Tables S1 and S2

Document S2. Article plus supplemental information
