# Supplementary material for: Multiplexed imaging and automated signal quantification in formalin-fixed paraffin-embedded tissues by ChipCytometry
Source: Cell Rep Methods. 2021 Oct 27;1(7):100104. doi: 10.1016/j.crmeth.2021.100104 (PMC9017205; doi:10.1016/j.crmeth.2021.100104)
Supplement: Document S1. Figures S1–S6 and Tables S1 and S2 [file mmc1.pdf]

Cell Reports Methods, Volume *1*

Supplemental information

Multiplexed imaging and automated signal quantification in formalin-fixed paraffin-embedded tissues by ChipCytometry

Sebastian Jarosch, Jan Köhlen, Rim S.J. Sarker, Katja Steiger, Klaus-Peter Janssen, Arne Christians, Christian Hennig, Ernst Holler, Elvira D'Ippolito, and Dirk H. Busch

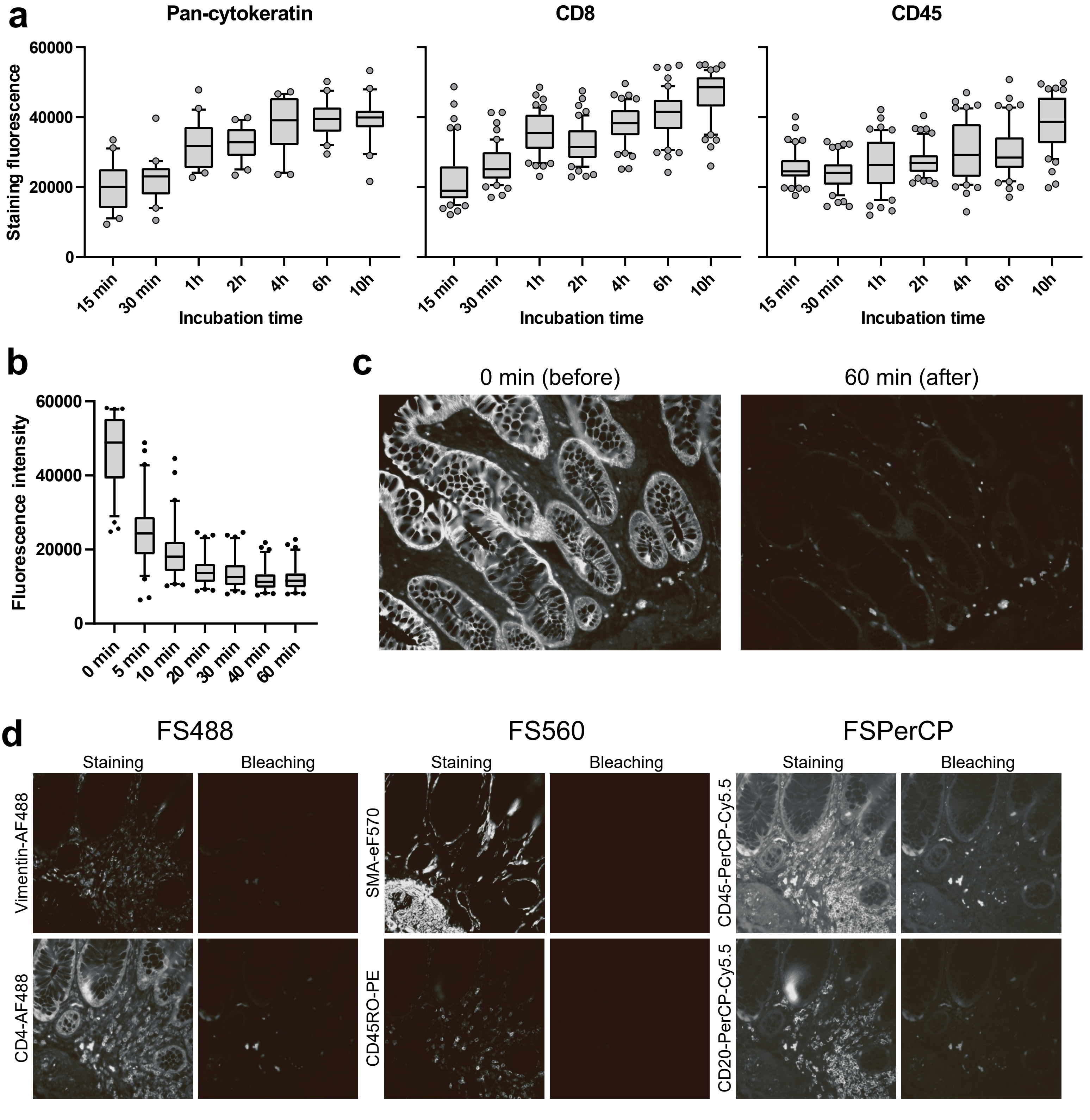

Figure S1. **Effects of antibody incubation and chemical bleaching on staining intensities (related to Figure 1).** FFPE healthy colon tissue sections were mounted on ChipCytometry chips, pre-treated with antigen retrieval buffer and Sudan Black B solution, and stained with fluorophore-conjugated antibodies as indicated. **a)** Tissue sections were incubated with the same antibody mix and analyzed after the indicated incubation times at 4°C. **b)** Tissue sections were incubated with fluorophore inactivation buffer (PBS, 24 mM NaOH, 4,5% H_2O_2) at the indicated incubation times. Images were acquired and fluorescence intensity quantified for each crypt. **c)** Representative images before and after fluorophore inactivation. **d)** Tissues sections were stained with two rounds of antibody cocktails. Chemical bleaching was performed between the two cycles. Showed are representative images before and after chemical bleaching, showing the feasibility of the method in ChipCytometry HMTI. In a) and b) data are depicted as interquartile ranges, with whiskers extending to 10% and 90% and outliers plotted as dots.

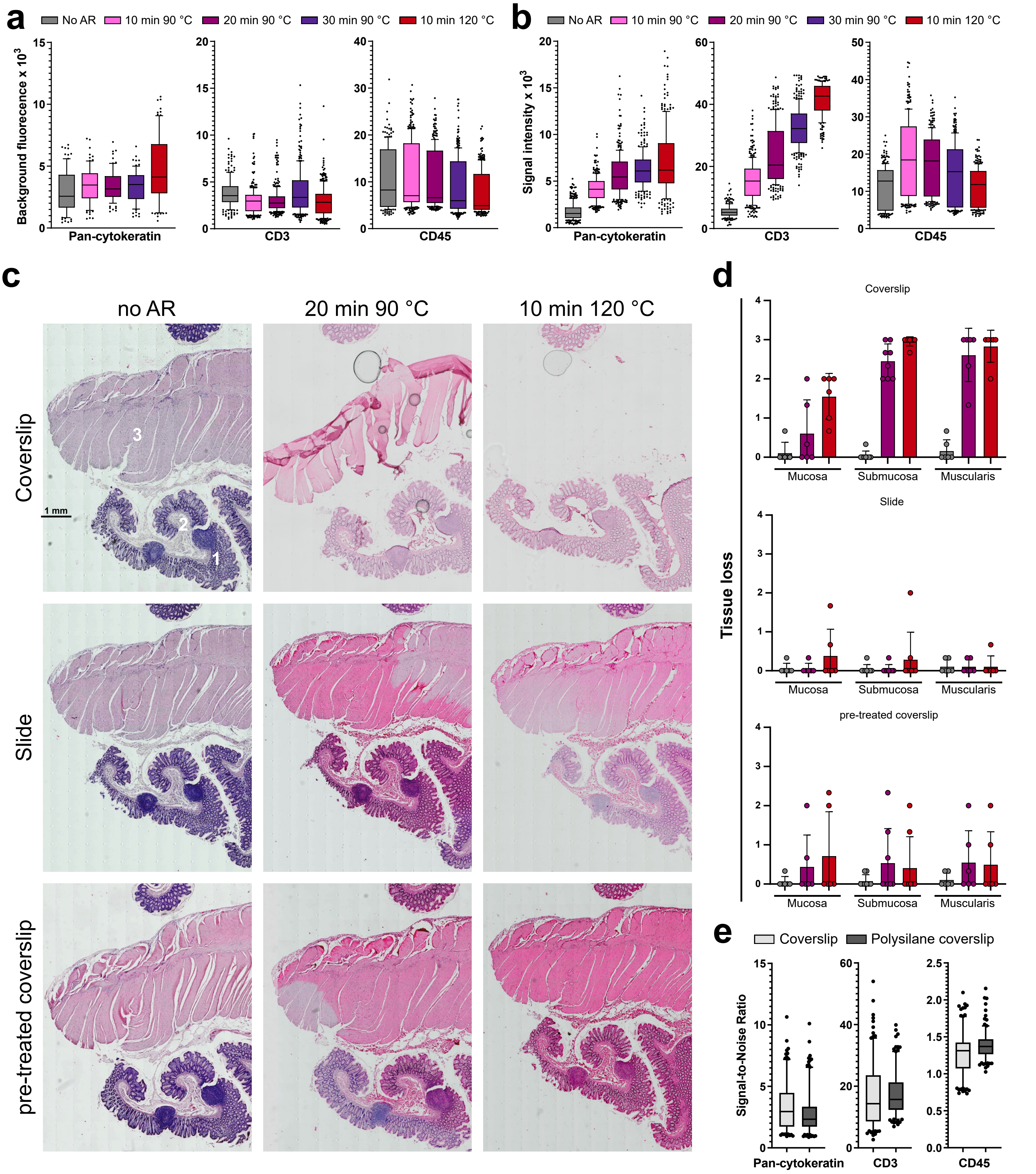

Figure S2. Effects of different antigen retrieval methods and coverslips on tissue integrity and staining intensity (related to Figure 1). a-b) Healthy colon tissues were treated with different antigen retrieval (AR) methods and background images were acquired prior staining. Background (a) and signal (b) intensities were quantified for individual crypts (pan-cytokeratin staining) or cells (CD3/CD45 staining) for 8 positions per section. Data are depicted as interquartile ranges, with whiskers extending to 10% and 90% and outliers plotted as dots of 3 donors from 2 independent experiments. **c-e)** Healthy colon tissues were mounted on different types of coverslips/slides and treated with different AR methods. **c)** Hematoxylin and Eosin staining of consecutive tissue sections. Numbers indicate regions of mucosa (1), submucosa (2) and muscularis (3). **d)** Scoring of tissue loss (0 = no tissue integrity loss, 3 = complete loss of the tissue). Data are shown as mean ± standard deviation. **e)** Quantification of signal-to-noise ratio for 20 min 90 °C antigen retrieval comparing conventional and pre-treated coverslips. Data are depicted as interquartile ranges, with whiskers extending to 10% and 90% and outliers plotted as dots. Individual crypts (pan-cytokeratin staining) or cells (CD3/CD45 staining) were quantified for 8 positions per section.

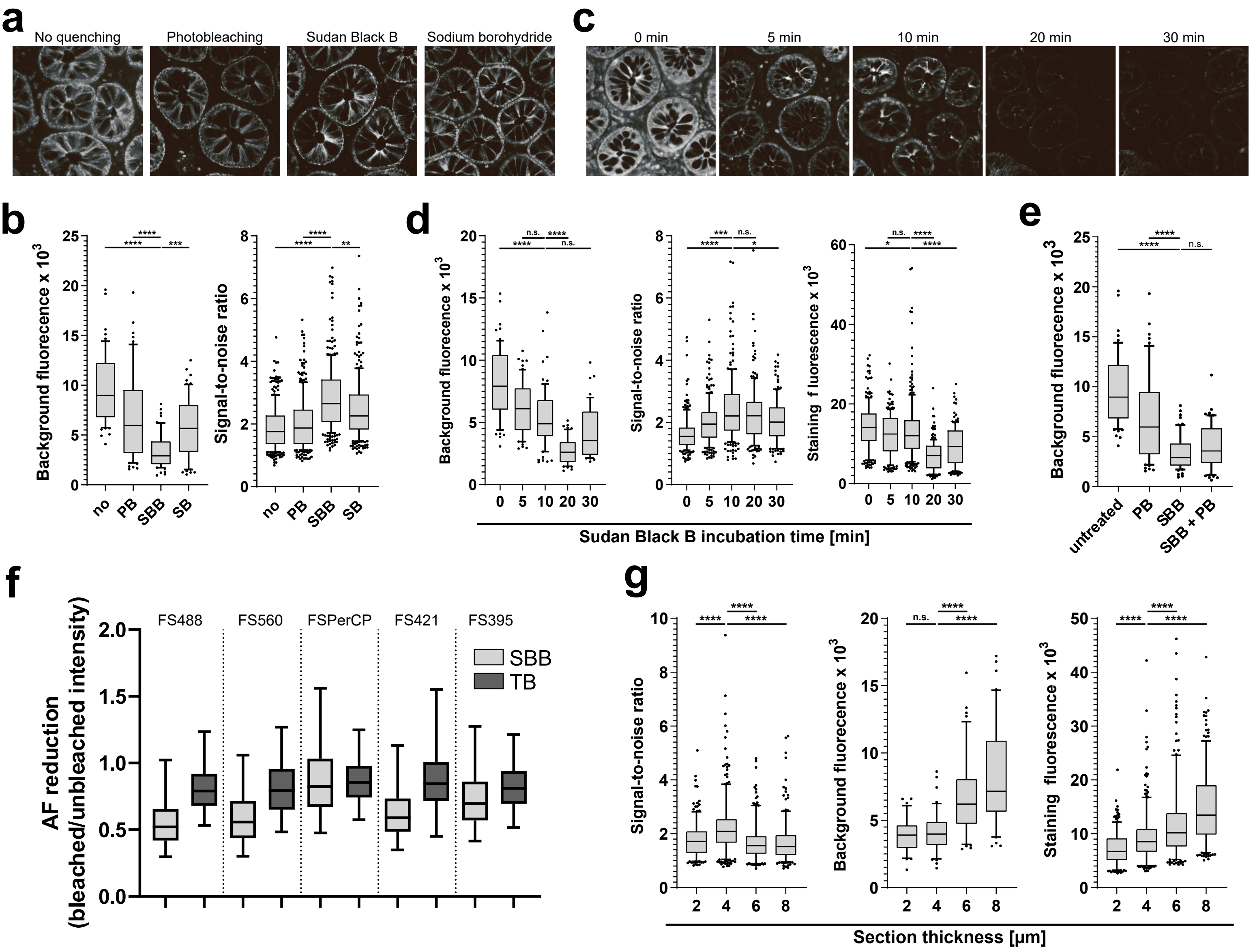

Figure S3. **Selection of treatment for optimal autofluorescence reduction (related to Figure 1). a-b)** Healthy colon sections received different treatments for autofluorescence reduction, and images showing crypt autofluorescence were acquired (a). Background (dark areas) and signal (pan-cytokeratin staining) intensities were quantified. Signal-to-noise ratio was calculated as crypt staining/background ratio (b). **c-d)** Healthy colon sections were treated with Sudan Black B solution for different incubation times. Representative images of crypt autofluorescence (c) and quantification of background (black areas), staining fluorescence (pan-cytokeratin staining) and signal-to-noise ratio (d) are shown. **e)** Combinatory effect was tested for Sudan Black B and photobleaching treatment. **f)** Comparison of bleaching capacity between Sudan Black B and True Black. **g)** Healthy colon samples were cut at different section thickness, pre-treated with Sudan Black B solution for 10 minutes and stained for pan-cytokeratin. Non-epithelial tissue parts were quantified as autofluorescence background. Background, staining fluorescence and signal-to-noise were quantified and calculated for the different section thicknesses. In figure b-d), f) and g) , data are pooled from 3 donors and 2 independent experiments, and depicted as interquartile ranges (whiskers extending to 10% and 90% and outliers plotted as dots). 8 positions per section were analyzed. Statistical testing was conducted by Tukey's test followed by Dunn's multiple comparisons test (*p<0.05; **p<0.01; ***p<0.001; ****p<0.0001). PB = Photobleaching, SBB = Sudan Black B, SB = Sodium borohydride, TB =True Black.

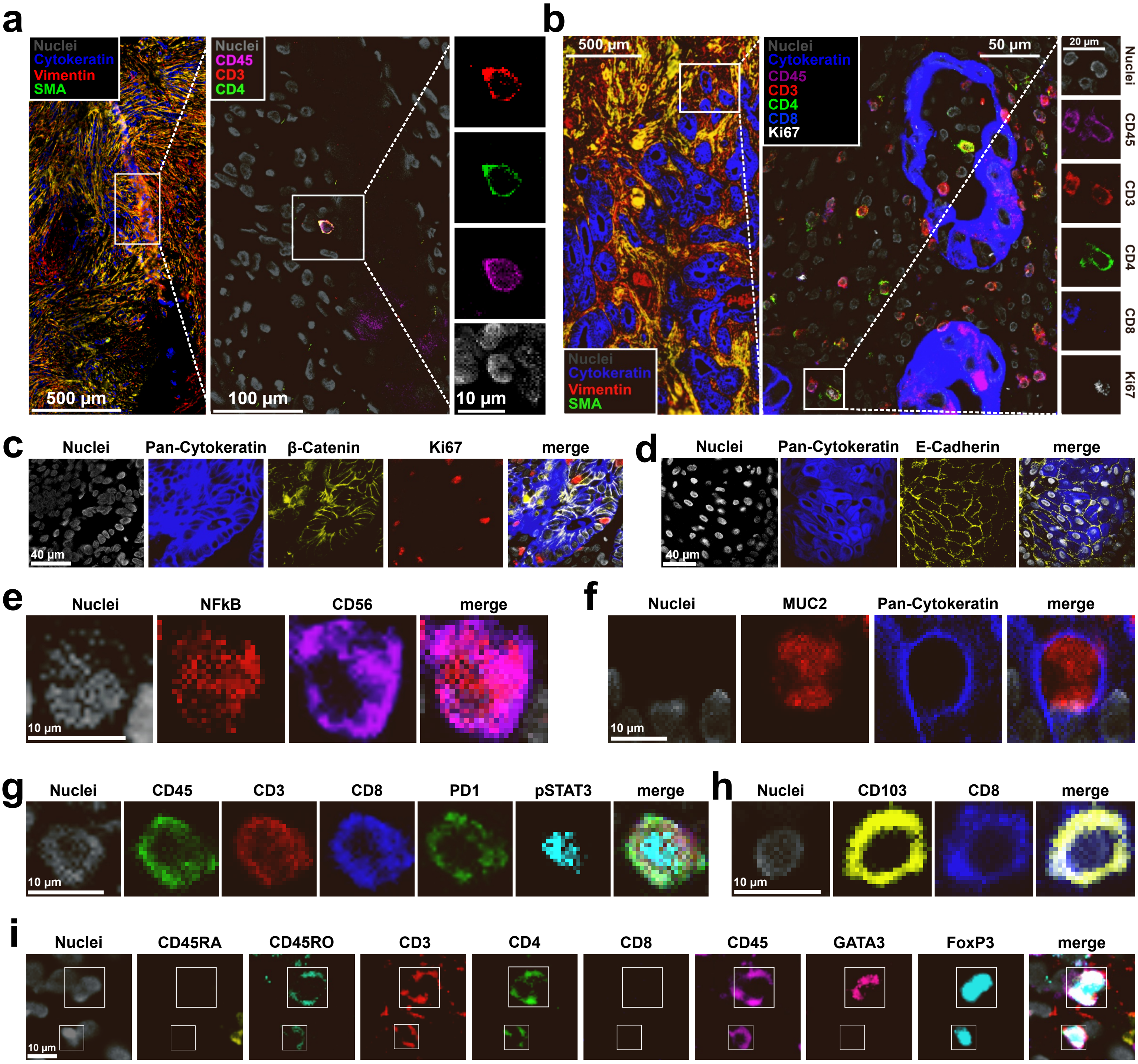

Figure S4. High-quality multiplexed staining of FFPE tissues with ChipCytometry (related to Figure 2). a-b) Multiplex ChipCytometry overlay of a human breast tissue with low immune infiltrate (number of markers = 6) (a) and a colorectal cancer tissue (number of markers = 10) (b). **c-d)** Representative images of staining of epithelial markers (pan-cytokeratin, β-catenin and E-Cadherin) in cancer tissue sections. **e-i)** Individual cells from colorectal cancer tissue sections. **e)** NF-kB expressing NK cell (CD56+), **f)** MUC2 expressing goblet cell in the epithelial compartment, **g)** pSTAT3 expressing CD8+ T cell, **h)** CD103 positive CD8+ tissue resident memory (TRM) cell. **i)** GATA3+ (upper cell) and GATA3- (lower cell) regulatory T cells in an inflamed human colon tissue.

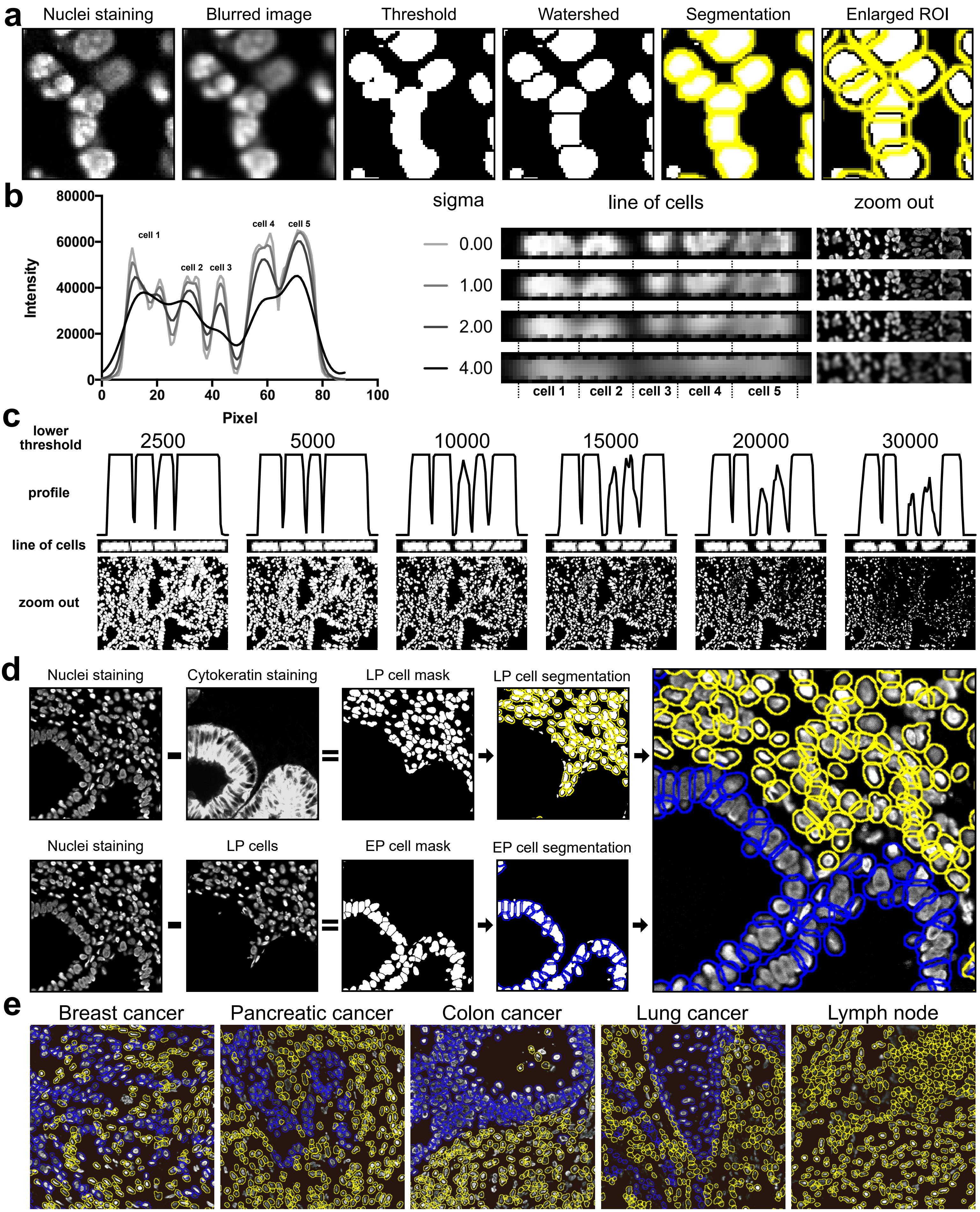

Figure S5. Cell segmentation via nuclei staining (related to Figure 3). a) Image series of a selected area from a nucleus staining depicting each step of the segmentation pipeline. **b)** The sigma value (intensity of blurring) was titrated. The intensity profile of 5 cells in a row (left) shows the separation of cells by the minima and maxima of the curves for different sigma values, with a clear smoothening of the curves with increasing sigma values. The corresponding images (middle) and the selected regions (right) depict visible differences between sigma values for the applied Gaussian blurring. A sigma value of 1 showed a good compromise between cell separation and reduction of intranuclear intensity differences. **c)** The watershed algorithm on the binary image allows, together with an increased lower threshold, to separate overlapping cells. Threshold titration shows how neighboring cells can be divided according to an optimal threshold value (10.000), above which proper cell segmentation is lost. **d)** Healthy colon tissues were stained with pan-cytokeratin. Either pan-cytokeratin staining (epithelial cells, EP) or nuclear staining of lamina propria (LP) cells was subtracted to the image prior to cell segmentation. **e)** Exemplary images of separate segmentation in different tissues types. Blue = segmented epithelial cells, yellow = segmented non epithelial cells.

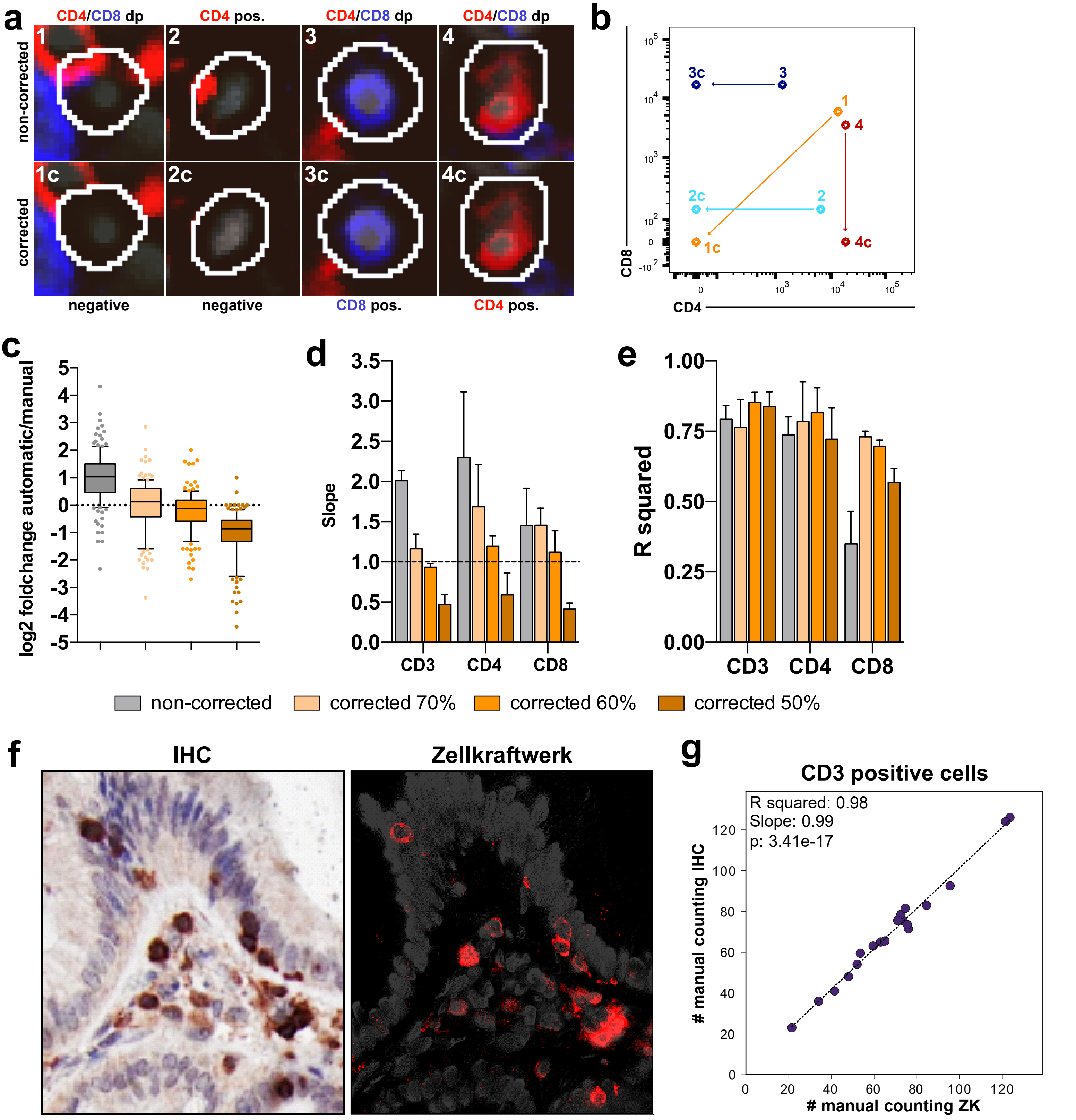

Figure S6. Titration of threshold value for optimal spatial spillover correction (related to Figure 3). a) Representative images to visualize the effect of spatial spillover correction. **b)** Cells shown in a) are plotted according to CD4/CD8 intensity values before (1-4) and after (1c-4c) spatial spillover correction. **c-e)** Cells positive for CD3, CD4 or CD8 staining were quantified via automated quantification according to different spillover thresholds or manual counting. Manual and automated cell counts were finally correlated. Shown are the fold change (c), the slopes (d) and the R squared values (e) for the correlation between automatic quantification with different spillover corrections vs. manual counting. **f)** Representative images of CD3 staining by ChipCytometry (left) and IHC (right) on the same tissue section. **g)** Correlation between manual quantification of ChipCytometry and IHC CD3 staining. Data in c) are depicted as interquartile ranges (whiskers extending to 10% and 90% and outliers plotted as dots) whereas data in d) and e) are expressed as mean ± SD for three donors.

Table S1. Human subject characteristics (related to STAR Methods)

	Description	Disease	Anatomical localization	Stage	Age	Sex
**1**	healthy adjacent tissue from colon resection	Adenocarcinoma	Rectum	TNM-stage (IUICC/AJCC): IIIB	70	male
**2**	healthy adjacent tissue from colon resection	Colon cancer	Colon	no information	66	male
**3**	healthy adjacent tissue from colon resection	Adenocarcinoma	Sigma	TNM-stage (IUICC/AJCC): IIA	77	female
**4**	biopsy from aHSCT patient	GvHD	Duodenum	Lerner Grade III	67	male
**5**	biopsy from aHSCT patient	GvHD	Caecum	Lerner Grade II	55	male
**6**	Colorectal cancer resection	adenocarcinoma	Caecum	TNM UICC-Klassifikation: T3, N0, M0. Stage IIA	85	male
**7**	Colorectal cancer resection	adenocarcinoma	Caecum	TNM UICC-Klassifikation: T3, N2a, M1a. Stage IVA	69	male
**8**	Colorectal cancer resection	adenocarcinoma	Colon ascendens	TNM UICC-Klassifikation: T3, N0, M0. Stage IIA	100	male
**9**	Colorectal cancer resection	adenocarcinoma	Caecum	TNM UICC-Klassifikation: T3, N0, M0. Stage IIA	81	female
**10**	Colorectal cancer resection	adenocarcinoma	Rectum	TNM UICC-Klassifikation: T3, N2b, M1. Stage IVA	75	Male
**11**	Pancreatic cancer resection	ductal adenocarcinoma	pancreas	TNM UICC-Klassifikation: T2, N1, M0, stage IIB	62	Male
**12**	Pancreatic cancer resection	ductal adenocarcinoma	pancreas head	TNM UICC-Klassifikation: T3, N2, M0, stage III	80	Male
**13**	Pancreatic cancer resection	ductal adenocarcinoma	pancreas	TNM UICC-Klassifikation: T3, N2, M0, stage III	71	Male
**14**	Pancreatic cancer resection	ductal adenocarcinoma	pancreas head	TNM UICC-Klassifikation: T4, N2, M0, stage III	67	Male
**15**	Pancreatic cancer resection	ductal adenocarcinoma	pancreas	TNM UICC-Klassifikation: T4, N2, M0, stage III	73	Male
**16**	Breast cancer resection	invasive lobular mammacarcinoma	Mammaabladat left	TNM UICC-Klassifikation: pT4b, pTis, pN3, M0. Stage IIIC	55	female

Table S2. Filter sets used for ChipCytometry (related to STAR Methods)

Filterset	Excitation	Emission	Filter Excitation	Filter Emission
FS395	364 nm – 366 nm	381 nm – 403 nm	365/2	392/23
FS421	370 nm – 410 nm	440 nm – 485 nm	390/40	460/50
FS488	450 nm – 490 nm	500 nm – 550 nm	470/40	525/50
FS560	525 nm – 575 nm	570 nm – 640 nm	550/25	605/70
FSPerCP	456 nm – 484 nm	672 nm – 748 nm	435/40	710/75
ALL (Bleach)	390 nm – 644 nm	-	364LP	
